# EEfinder, a general purpose tool for identification of bacterial and viral endogenized elements in eukaryotic genomes

**DOI:** 10.1016/j.csbj.2024.10.012

**Published:** 2024-10-18

**Authors:** Yago José Mariz Dias, Filipe Zimmer Dezordi, Gabriel da Luz Wallau

**Affiliations:** a Núcleo de Bioinformática, Instituto Aggeu Magalhães (IAM), Fundação Oswaldo Cruz (FIOCRUZ), Recife, PE, Brazil; b Departamento de Entomologia, Instituto Aggeu Magalhães (IAM), Fundação Oswaldo Cruz (FIOCRUZ), Recife, PE, Brazil; c Curso de Graduação em Biomedicina, Centro de Biociências, Universidade Federal de Pernambuco, Recife, PE, Brazil; d Department of Arbovirology and Entomology, Bernhard Nocht Institute for Tropical Medicine, WHO Collaborating Center for Arbovirus and Hemorrhagic Fever Reference and Research, Hamburg, Germany; e Programa de Pós Graduação em Biodiversidade Animal and Programa de Pós Graduação em Bioquímica Toxicológica, Universidade Federal Santa Maria (UFSM), Rio Grande do Sul, Brazil

**Keywords:** Comparative genomics, Symbionts, Integration, Genome evolution, Virus, Bacteria

## Abstract

Horizontal gene transfer is a phenomenon of genetic material transmission between species with no parental relationship. It has been characterized among several major branches of life, including among prokaryotes, viruses and eukaryotes. The characterization of endogenous elements derived from viruses or bacteria provides a snapshot of past host-pathogen interactions and coevolution as well as reference information to remove false positive results from metagenomic studies. Currently there is a lack of general purpose standardized tools for endogenous elements screening which limits reproducibility and hinder comparative analysis between studies. Here we describe EEfinder, a new general purpose tool for identification and classification of endogenous elements derived from viruses or bacteria found in eukaryotic genomes. The tool was developed to include six common steps performed in this type of analysis: data cleaning, similarity search through sequence alignment, filtering candidate elements, taxonomy assignment, merging of truncated elements and flanks extraction. We evaluated the sensitivity of EEfinder to identify endogenous elements through comparative analysis using data from the literature and showed that EEfinder automatically detected 97 % of the EVEs compared to published results obtained by manual curation and detected an almost exact full integration of a *Wolbachia* genome described using wet-lab experiments. Therefore, EEfinder can effectively and systematically identify endogenous elements with bacterial/viral origin integrated in eukaryotic genomes. EEfinder is publicly available on https://github.com/WallauBioinfo/EEfinder.

## 1 Introduction

Numerous viruses and bacteria are symbionts of eukaryotic species, living in close proximity and interacting in many different ways with their host cells. Besides cells and protein-protein interactions, these symbionts may also swap genes with eukaryotic species through horizontal transfer giving origin to Endogenous Viral Elements (EVEs) [1] and Endogenous Bacterial Elements (EBEs) [2]. This phenomenon of genetic material transfer between non-parental organisms is called Horizontal Gene Transfer (HGT) and is known to occur across all major branches of life [3]. Horizontally transferred elements can be vertically inherited if they integrate into the genome of germline cells [4]. EBEs and EVEs have been increasingly recognized as an important source of evolutionary novelties such as piRNAs produced from EVEs acting as an antiviral defense in mosquitoes and EBEs acting as a new sexual chromosome in *Armadillidium vulgare* 2, 5. Moreover, EVEs can be used as molecular fossils 1, 6, 7 to calibrate more precisely the evolutionary dating of viral families [6] owing to their lower substitution rate compared to cognate circulating viruses [6].

The endogenization of viral and bacterial sequences into eukaryotic host genomes is currently well-recognized but the exact endogenization mechanisms remain unclear. Transposable elements (TEs), which are abundant in eukaryotic genomes and are capable of self-replication and non-homologous recombination, may both contribute to viral endogenization [8]. Supporting this hypothesis, non retroviral Integrated RNA Virus Sequences (NIRVS) endogenization in mosquito genomes is often found in TE-rich regions [5]. Transposable Elements may also participate in the virus-TE double-strand RNA and DNA hybrid biogenesis, which may facilitate integrase recognition and integration of these chimeric molecules into the eukaryotic genome [9]. Alternatively, viral and bacterial genetic material may also integrate into host eukaryotic genomes through non-homologous end joining repair mechanisms [10]. After integration, EVEs and EBEs may accumulate mutations and shatter through time or remain conserved evolving under purifying selection depending upon the fitness impact on the host [4]. For instance, the syncytin gene, which mediates placental cytotrophoblast fusion, shares 100 % identity with the Human endogenous retrovirus-W (HERV-W) [11], this syncytin gene was likely integrated into the ancestor of all placental mammals around 25 Mya [12]. On the other hand, Leclercq et al. identified a 3 MB single insertion of a *Wolbachia* bacteria genome in the *Armadillidium vulgare* host genome that is shattered in several in tandem small and large genomic fragments, this whole bacterial genome insertion gave rise to a new sex chromosome [2].

The correct identification of EVEs and EBEs can not only further our understanding of the co-evolution between hosts and symbionts but can also have direct implications on the identification of true viral/bacterial infection from endogenized viral/bacterial genomic fragments. After endogenization, these elements may transcribe RNA molecules that may be wrongly identified as generated by non-integrated bona fide viral/bacterial genomes leading to false positives in metagenomics studies 9, 13. Similarly, endogenous elements from *Wolbachia* genomes can bias studies of this group of bacteria in different eukaryotes [14]. Therefore, EVEs and EBEs systematic identification and reporting is important to correctly ascertain the integrated or non-integrated nature of the symbiont's genomic molecules detected.

Different studies 9, 15, 16 employ a series of steps for EVEs and EBEs characterization such as: small contigs/scaffolds removal to avoid identifying symbiont contigs contaminants as endogenous elements [16]; alignment between proteins derived from the host genome and viral proteins to increase sensitivity 9, 15; filtering out potential wrongly identified endogenous elements by comparing them with host proteins 9, 15; merging nearby endogenous elements loci belonging to the same taxon, as they may represent shattered sequences of an original endogenization [17]; and the assessment of the flanking regions surrounding the elements for the presence of host genes or TEs 9, 15. Besides the existence of tools and workflows for specific host genomes 18, 19, 20 or viral families [17], up to date there is no general-purpose tool available that integrate most or all these steps, and typically some level of manual curation is applied in these studies, leading to disparate results even considering the same genome version for a given species [15]. This lack of standard procedures with minimal or no manual intervention hinder comparison between studies and more broad inferences about EVEs and EBEs.

Therefore, there is a clear need for an automated approach to precisely identify and characterize EVEs or EBEs to ultimately allow reproducibility in this research field and the differentiation of these integrated elements and viral and bacterial non integrated DNA. Here we developed EEfinder, a Python package and Command Line Interface (CLI) tool for systematic identification and characterization of viral/bacterial endogenous elements in eukaryotic genomes.

## 2 Materials and methods

### 2.1 EEfinder development

**Fig. 1:**
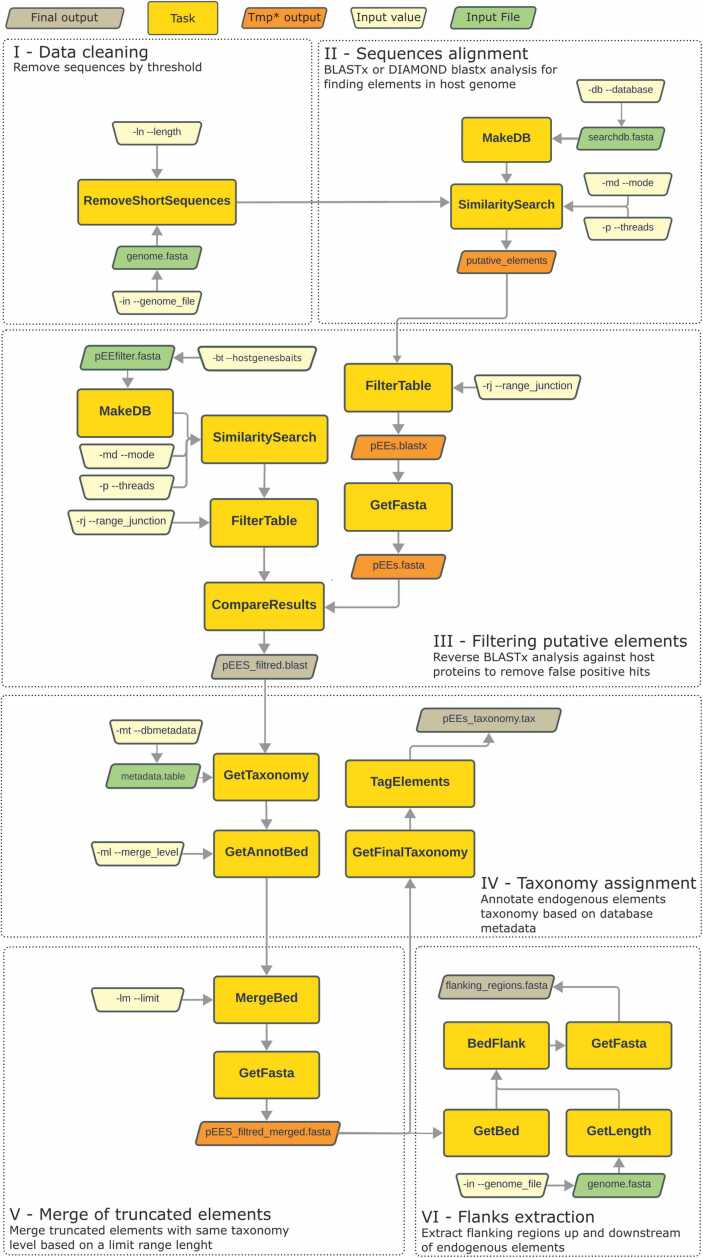
**:** EEfinder six steps from data input/cleaning to taxonomy assignment and final sequence extraction. *Temporary output files. With default settings, the temporary outputs are deleted, but the user can set an option to keep those files.

EEfinder was developed in Python 3.9 and incorporates six main steps commonly applied in endogenous elements genomic characterization 5, 9, 15, 21. It requires four input files to work (Fig. 1 and **SupplementaryTable1**): a multifasta file containing the genomic sequences, a multifasta file of virus/bacterial protein database, a metadata table with taxonomic annotation fields (accession id, species, genus, family, molecule type, protein product, host), and a host genes baits fasta file with host gene proteins to filter out false positives endogenous elements.

### 2.2 Data cleaning

A filter for small genomic sequences was implemented by setting a minimum contig/scaffold length used in the similarity search step (Fig. 1**-I**). This approach is used in other studies 5, 16 to remove genome assembly contamination that may hinder additional analysis that requires flanking sequences to validate true EEs.

### 2.3 Similarity search

EEfinder was developed with two pairwise-alignment algorithms: BLAST v2.5.0 [22] and DIAMOND 2.0.15 [23], allowing the user to choose between them (Fig. 1**-II**). Both methods are implemented to run blastx analysis (proteins translated from the eukaryotic genome against viral or bacterial proteins) considering that comparison between protein sequences are more sensitive than nucleotide alignments 24, 25. Since endogenous elements can be highly divergent from the cognates viral and bacterial genomes 1, 6, 7 we implemented the BLOSUM45 matrix in the blastx analysis and the word_size 3 to increase the protein alignments sensitivity.

### 2.4 Putative elements filter

Some bacterial or viral proteins share ancient common ancestors with eukaryotic proteins (e.g., DNA polymerases) 9, 15. Therefore, putative elements identified through pairwise alignment require extra analysis steps to filter out false positives using eukaryotic proteins databases. This step is normally performed manually 9, 15 comparing the potential EEs and host proteins alignment score. EEfinder automates this process by running a reverse pairwise alignment against the host protein database using the host genes baits file (Fig. 1**-III**). Through the comparison of the initial alignment and the reverse alignment, EEfinder retains only those elements with higher bitscores against the bacterial/viral database than the host proteins database. We have chosen the bitscore to compare the results because this metric is not influenced by the size of the database ensuring more consistent results between analysis [26].

### 2.5 Taxonomic assignment

In the next step, the EEfinder uses the virus/bacterial metadata table provided by the user to transfer the taxonomic information of each EE based on the most closely related viral/bacterial protein (Fig. 1**-IV**). The tool documentation has instructions of how to build this file and also includes an additional script for automating the table building if the user data is provided.

### 2.6 Merging fragmented elements

Endogenous element integrations are usually shattered over evolutionary time by the accumulation of mutations and/or transposable elements insertions [4] as well as a result from defective viral particle integration [27] appearing as fragmented EEs loci. There is no standard threshold for nucleotide base range or taxonomic level to merge fragmented elements. The EEfinder has parameters that allow the user to merge cis elements identified by the same viral/bacterial family or genus (taxon defined by the user) that are apart from a specific nucleotide bases range (Fig. 1**-V, SupplementaryFigure 1**). This parameter can be fully set by the user allowing no merging to various nucleotide ranges and taxonomy levels merging.

### 2.7 Flanks extraction

In the final step, EEfinder extracts flanks of each element boundaries, with the length adjustable by the user (Fig. 1**-VI)**. These flanks can be utilized in further analyses, such as gene orthology, TEs searches, or host gene identification 15, 28.

### 2.8 Alignment tools benchmark

To compare the use for each pairwise-alignment mode (BLAST and DIAMOND), we performed tests using different alignment modes: BLASTx and DIAMOND blastx on fast, sensitive, mid-sensitive, more-sensitive, very-sensitive and ultra-sensitive modes. After that, we compared the number of EVEs retrieved, the identity range and the time to run each analysis.

### 2.9 Computational resources usage

To determine the computational resources demand for EEfinder analysis, we ran additional tests with bacterial and virus databases with limited computational resources. We configured four test groups with varying thread counts and corresponding RAM memory: 4 threads with 8 GB, 8 threads with 16 GB, 16 threads with 32 GB, and 32 threads with 64 GB. Each test was conducted three times to estimate the variability, mean and standard deviation.

### 2.10 Endogenous viral elements validation

To benchmark EEfinder for EVEs screening, we compared the results with the study of Whitfield et al. [15], this study was chosen once it provides some automated scripts that are widely used in other studies 29, 30. EVEs screening was performed on the Aag2 genome GCA_021653915 from *Aedes aegypti* (**SupplementaryTable1**). Two viral protein databases were employed: all viral proteins available on NCBI Virus RefSeq (updated September 8, 2022) and proteins described in the study by Whitfield et al. [15]. To compare EEfinder results with those from Whitfield et al., we updated the taxonomy of EVEs identified by Whitfield et al. using BLASTx analysis against the NCBI Virus RefSeq proteins. The host protein database was built by downloading all *Ae. aegypti* proteins available on NCBI RefSeq (updated September 8, 2022).

To assess the impact of hypothetical/uncharacterized proteins on the putative EVEs filter, we created two versions of this database: one without filtering “hypothetical” or “uncharacterized” proteins and another excluding all proteins labeled as “hypothetical” or “uncharacterized”. The results comparison were acquired with the Whitfield described proteins database and utilizing the merge level (-ml) set to "Family". To verify the results differences between Whitfield *et. al.* and EEfinder we used a set of in-house scripts (**SupplementaryTable2,** see **Data Availability** section), that analyzed if the start and end position of each element overlapped with a maximum 100 nt of difference.

In order to check if Whitfield et al. described fragmented EVEs as separate endogenization events, we extracted the ORFs of each element using ORFfinder [31] and aligned against the closest viral gene with MAFFT v7 [32], verifying if the fragmented ORFs from EVEs displayed continuity in respect to the reference protein from the closest viral homologous region. This process was done for two cases for each family that displayed overlapping elements merged by EEfinder (**SupplementaryFigure2**).

### 2.11 Endogenous bacterial elements sensitivity validation

To evaluate the applicability of EEfinder for endogenous bacterial elements detection, we aimed to reproduce the results from Leclercq et. al [2] that described the insertion of a bacteria of the genus *Wolbachia* (wVulC) found integrated in the genome of a pill bug *Armadillidium vulgare* (GCA_001887335). The data used for tests are described in **SupplementaryTable1**. We utilized the WvulC strain as a bacterial database, this strain showed in the Leclerq et al. manuscript to be the most similar *Wolbachia* genome to the endogenized one. The following reasoning was used in this analysis:

We tested different parameters for limit merge (-lm) and range junction (-rj). For the nucleotide bases range, 10000 and 100 bases were optimal to recover results similar to the Leclercq study. The merge level (-ml) was set to genus because we used a database with only proteins of the *Wolbachia* genus, hence we do not expect to detect any differences using other options on this parameter. After the analysis, we compared the results using the range between flaking EEs as well as the length of the element.

## 3 Results and discussion

### 3.1 EEfinder

The tool is available at (https://github.com/WallauBioinfo/EEfinder) and documented in a wiki page including installation, dependencies versions, testing - with test dataset included in the repository - and documentation about different arguments as well as a short guide to build the input files of the tool. The EEfinder is currently in the first production version (v1.0.0) following the semantic versioning specification [33]. All tests described in the following sections are performed using the EEfinder v1.0.0.

### 3.2 Benchmark of alignment tools

**Fig. 2:**
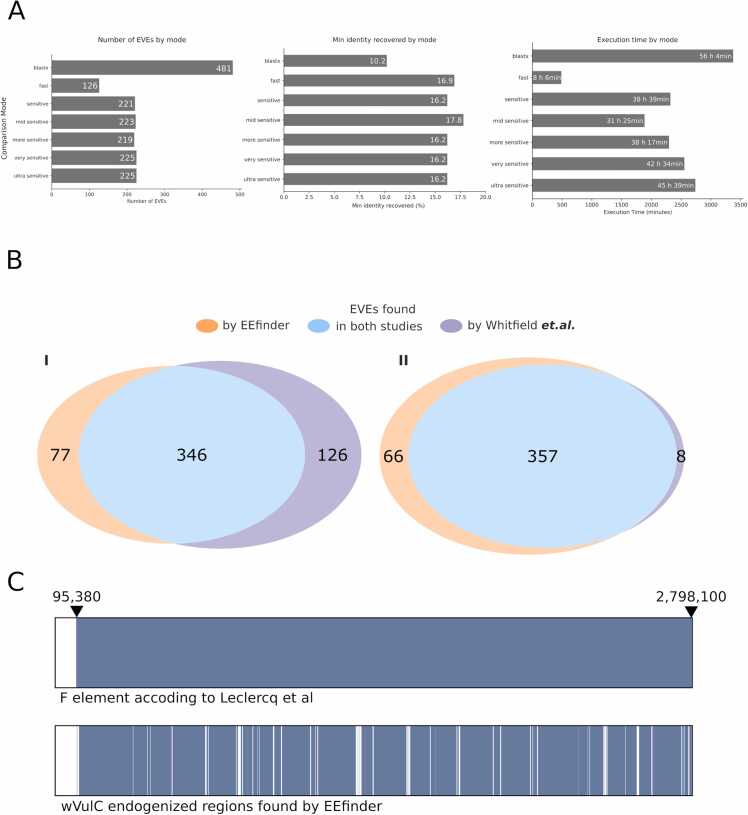
**:** EEfinder benchmark alignment tools and validation. **A.** BLASTx and DIAMOND methods comparrison displaying number of EVEs recovered, minimum identity and execution time using different methods set up. **B.** Comparison between EEfinder and Whitifiled *et. al.*[15] regarding the number of EVEs recovered by each strategy and the overlapping results. **B-I.** Comparison without merging of fragmented elements. **B-II.** Comparison with fragmented elements merged by EEfinder. **C.** Region of endogenized wVulC identified by Leclercq *et. al.*[2] and EEfinder on the scaffold 1 of *A. vulgare*. The matched regions found in each study are displayed in blue, the absence of a *Wolbachia* match is represented in white.

Between the DIAMOND modes, very sensitive mode had the best sensibility, recovering 225 EVEs showing an identity range of 16.2 % to 100 %. This analysis took 42 h and 34 min to finish. The fast mode retrieved 126 elements with identity range varying from 16.9 % to 100 % in 8 h and 6 min. Although BLAST showed higher sensitivity with 481 elements showing identity ranging between 10.2–100 %, it took 56 hours and 14 min to finish (Fig. 2**A**). In the original DIAMOND manuscript [23], the authors stated that the use of a reduced amino acid alphabet does not alter sensitivity, but we showed a clear impact on sensitivity on highly divergent sequences normally found in EVEs studies [9].

### 3.3 Computational resources usage

The shortest processing time in the resources benchmark test was using 8 threads and 16 GB of memory with a mean of 2 h and 20 min for the virus dataset, meanwhile, the longest time (mean of 2 h and 38 min) was using 4 threads and 8 GB of memory (Table 1).

**Table 1:** Computational resources comparison of EVEs and EBEs analysis on different computation resource configurations. *Time in minutes. ᐤResults obtained using Whitfield's EVEs proteins as queries against the *Aedes aegypti* Aag2 genome. ⋆Results obtained using wVulC proteins against the *Armadillidium vulgare* genome (GCA_001887335).

**Dataset**	**Number of threads**	Test 1 *	Test 2 *	Test 3 *	Mean*	**Standard Deviation**
Viralᐤ	4	150	173	151	158	0.009
Viralᐤ	8	140	135	145	140	0.003
Viralᐤ	16	166	140	153	153	0.009
Viralᐤ	32	145	159	160	2.07	0.006
Bacterial⋆	4	2.2	2	2	1.5	0
Bacterial⋆	8	1.4	1.5	1.6	1.3	0
Bacterial⋆	16	1.3	1.3	1.3	1.3	0
Bacterial⋆	32	1.2	2.3	2.5	2	0

For the bacterial dataset, we had the lowest processing time (1 min and 18 s) with 16 threads and 32 GB of RAM. The longest time was with 4 threads and 8 GB of memory, totalizing 2 min and 4 s (Table 1). In conclusion, EEfinder does not require significant computational resources and hence it is suitable to be used even on personal computers.

The tool comes with two algorithms for similarity analysis that can be used for different purposes, BLASTx with the highest sensitivity for more comphrehensive identification of EE in a genome. On the other hand, DIAMOND has a lower sensitivity with a lower runtime; these optional features allow more flexibility to the end user.

### 3.4 EVEs screening

In our first test with the refseq including “uncharacterized” or “hypothetical” proteins in the host genes baits database against NCBI virus refseq proteins, we detected 377 EVEs (**SupplementaryTable3**). On the other hand, in the tests with the same host genes baits database except for uncharacterized and hypothetical proteins, we retrieved 578 elements. When we use only the EVEs proteins described by Whitfield et al. (472 EVEs elements) [15] we were able to characterize 423 elements. It is likely that these “uncharacterized” and “hypothetical” proteins were actual unclassified EVEs (EVEs with no taxonomy) misclassified/misannotated as unknown host proteins. These results reinforce the necessity to take into consideration a more detailed annotation of EVEs found in eukaryotic genomes. Furthermore, it is important to initiate a review effort for those host protein databases in order to reclassify these EVE proteins that lack annotation.

In order to validate if merging flanking elements with the same taxonomic assignment is biologically meaningful, we extracted the ORFs of both studies and aligned them with MAFFT v7 software [32] against the best match protein. The validation process was done with 6 merging events (**SupplementaryFigure3–4**). We were able to show that in three distinct examples (*Blueberry necrotic ring blotch virus*, *Citrus Leprosis* and *Shayang Fly Virus 1)* Whitfield and collaborators identified a single element as multiple independent endogenization events while EEfinder correctly merged these EVE loci into a single locus (**SupplementaryFigure4**). It is noteworthy to point out that EEfinder merged elements in which ORFs were arranged in sequence, one after another, suggesting that these elements were likely derived from a single integration/endogenization event (**SupplementaryFigure4**).

More broad comparison of EVEs loci found by Whitfield and EEfinder resulted in 346 elements found by both analyses, while 126 elements were exclusively detected by Whitfield and 77 exclusive elements were detected by EEfinder (Fig. 2**B-I**). Considering the curated merged EEfinder dataset, there are 357 common elements in the two analyses, with 8 exclusive elements detected by Whitfield and 66 exclusive elements detected by EEfinder (Fig. 2**B-II**).

The EVEs found belong to three most abundant families: Rhabdoviridae, Flaviviridae and Chuviridae. Of the 13 families detected, 5 were characterized only by EEfinder: Spinareoviridae, Virgaviridae, Sedoreoviridae, Closteroviridae and Bromoviridae (**SupplementaryFigure5**). Whitfield had 1 exclusive family (Reoviridae). The number of elements in EEfinder followed the same distribution of Whitfield et al. analysis (**SupplementaryFigure5**). The proteins retrieved from EEfinder vary in length between 500 to 2000 pb (**SupplementaryFigure6**). The family Kitaviridae showed many elements with length superior to 2000 pb, with an element larger than 5000 pb.

The total bases retrieved in each study were similar, with a difference of 79.189 bp (**SupplementaryTable5**). The total of bases retrieved by EEfinder was 405,550 pb and Whitfield *et. al.* identified 326,364 bp. The results were consistent between Whitfield and our study regarding the overall EVE content in the Aag2 genome. Moreover, the merging of elements by EEfinder could potentially yield more precise results with improved biological significance.

### 3.5 EBEs screening

Comparing our results with Leclercq’s study [2], the search against the isopod *Armadillidium vulgare* genome using the *Wolbachia* wVulC proteins resulted in multiple in tandem hits in scaffold 1 that corroborates the single endogenization event with the same start and end loci position defined by Leclercq 95,380 - 2798,100 (Fig. 2**C**). Even with the fragmentation displayed by EEfinder, the tool could detect the integration event showing its applicability to identify EBEs in a reproducible way. But the Leclercq *et. al.* study did not clarify if the element found is fragmented as shown by EEfinder. This event may be a result of the accumulation of mutations (SNPs and indels) after the integration event.

### 3.6 Limitations

The EEfinder was developed as a general purpose tool to identify viral or bacterial elements endogenized in eukaryotic genomes based on similarity analysis, so the results are highly dependent on the information present on virus and baits databases parsed by the user. Moreover, we did not include any step to filter TEs - owing to the plethora of strategies that can be used and their variable impact onto TEs identification [34] - which means that the use of EEfinder for Endogenous Retroviruses, or viral taxa with a history of coevolution with transposon proteins can be detected by EEfinder, but follow up and more in depth analysis are necessary to ascertain the viral origin and precisely access the elements boundaries and link/fusion with endogenous transposable elements.

## 4 Conclusion

In this study, we developed the first general-purpose automated tool for the identification of endogenous elements in eukaryotic genomes. The EEfinder sensitivity validation tests have proven the tool's capability to find endogenous elements through replicating results available in the literature for EVEs and EBEs. Moreover, EEfinder has low computational requirements, it can be used even in low-end personal machines. This new tool is expected to enhance the reproducibility of future studies contributing to more broadly and comparable EEs analysis in a wide range of eukaryotic genomes and fostering the construction of databases for refining metagenomic results.

## Funding source declaration

G.L.W. is supported by the Conselho Nacional de Desenvolvimento Científico e Tecnológico (CNPq) through their productivity research fellowships (307209/2023–7). This research received funding for a undergraduate scholarship of Y.J.M.D. from Fundaç ão de Amparo à Ciência e Tecnologia do Estado de Pernambuco (FACEPE).

## CRediT authorship contribution statement

**Gabriel da Luz Wallau:** Writing – review & editing, Writing – original draft, Visualization, Validation, Supervision, Resources, Project administration, Methodology, Investigation, Funding acquisition, Conceptualization. **Yago José Mariz Dias:** Writing – review & editing, Writing – original draft, Visualization, Validation, Methodology, Investigation, Formal analysis, Data curation. **Filipe Zimmer Dezordi:** Writing – review & editing, Writing – original draft, Visualization, Validation, Supervision, Software, Resources, Methodology, Investigation, Formal analysis, Data curation, Conceptualization.

## Declaration of Competing Interest

None.

## Data Availability

A jupyter notebook with all python scripts for data treatment (intersec.ipynb) and all lines of codes used in this study (analysis.ipynb) along with the source code of the EEfinder can be found at Figshare: https://doi.org/10.6084/m9.figshare.25864525.v2. The branch of EEfinder with the current version of the code can be found here: https://github.com/WallauBioinfo/EEfinder. The NCBI accession code of the genomes used in this study: *Aedes aegypti* Aag2 GCA_021653915 and *Armadillidium vulgare V1* GCA_001887335.1.

## References

[1] Katzourakis A., Gifford R.J. Endogenous Viral Elements in Animal Genomes. Malik HS, ed. *PLoS Genet*. 2010;6(11):e1001191. doi:10.1371/journal.pgen.1001191. 10.1371/journal.pgen.1001191 PMC2987831 21124940

[2] Leclercq S., Thézé J., Chebbi M.A. (2016). Birth of a W sex chromosome by horizontal transfer of *Wolbachia* bacterial symbiont genome. Proc Natl Acad Sci USA.

[3] Holmes E.C. (2011). The evolution of endogenous viral elements. Cell Host Microbe.

[4] Aswad A., Katzourakis A. (2012). Paleovirology and virally derived immunity. Trends Ecol Evol.

[5] Palatini U., Miesen P., Carballar-Lejarazu R. (2017). Comparative genomics shows that viral integrations are abundant and express piRNAs in the arboviral vectors Aedes aegypti and Aedes albopictus. BMC Genom.

[6] Katzourakis A. (2013). Paleovirology: inferring viral evolution from host genome sequence data. Philos Trans R Soc B.

[7] Koutsovoulos G., Makepeace B., Tanya V.N., Blaxter M., Feschotte C. (2014).

[8] Tassetto M., Kunitomi M., Whitfield Z.J. (2019). Control of RNA viruses in mosquito cells through the acquisition of vDNA and endogenous viral elements. eLife.

[9] Dezordi F.Z., Vasconcelos C.R. dos S., Rezende A.M., Wallau G.L. (2020). In and outs of chuviridae endogenous viral elements: origin of a potentially new retrovirus and signature of ancient and ongoing arms race in mosquito genomes. Front Genet.

[10] Husnik F., McCutcheon J.P. (2018). Functional horizontal gene transfer from bacteria to eukaryotes. Nat Rev Microbiol.

[11] Mi S., Lee X., Li X. ping (2000). Syncytin is a captive retroviral envelope protein involved in human placental morphogenesis. Nature.

[12] Bannert N., Kurth R. (2006). The evolutionary dynamics of human endogenous retroviral families. Annu Rev Genom Hum Genet.

[13] Lara Pinto A.Z.D., Santos De Carvalho M., De Melo F.L., Ribeiro A.L.M., Morais Ribeiro B., Dezengrini Slhessarenko R., Schneider B.S. (2017).

[14] Inácio da Silva L.M., Dezordi F.Z., Paiva M.H.S., Wallau G.L. (2021). Systematic review of wolbachia symbiont detection in mosquitoes: an entangled topic about methodological power and true symbiosis. Pathogens.

[15] Whitfield Z.J., Dolan P.T., Kunitomi M. (2017). The diversity, structure, and function of heritable adaptive immunity sequences in the aedes aegypti genome. Curr Biol.

[16] Flynn P.J., Moreau C.S. (2019). AssesSing the Diversity of Endogenous Viruses throughout Ant Genomes. Front Microbiol.

[17] Vassilieff H., Haddad S., Jamilloux V. (2022). CAULIFINDER: a pipeline for the automated detection and annotation of caulimovirid endogenous viral elements in plant genomes. Mob DNA.

[18] Garazha A., Ivanova A., Suntsova M. (2015). New bioinformatic tool for quick identification of functionally relevant endogenous retroviral inserts in human genome. Cell Cycle.

[19] Tongyoo P., Avihingsanon Y., Prom-On S., Mutirangura A., Mhuantong W., Hirankarn N., Belshaw R. (2017).

[20] Tokuyama M., Kong Y., Song E., Jayewickreme T., Kang I., Iwasaki A. (2018). ERVmap analysis reveals genome-wide transcription of human endogenous retroviruses. Proc Natl Acad Sci USA.

[21] Russo A.G., Kelly A.G., Enosi Tuipulotu D., Tanaka M.M., White P.A. (2019). Novel insights into endogenous RNA viral elements in Ixodes scapularis and other arbovirus vector genomes. Virus Evol.

[22] Camacho C., Coulouris G., Avagyan V. (2009). BLAST+: architecture and applications. BMC Bioinforma.

[23] Buchfink B., Reuter K., Drost H.G. (2021). Sensitive protein alignments at tree-of-life scale using DIAMOND. Nat Methods.

[24] Abascal F., Zardoya R., Telford M.J. (2010). TranslatorX: multiple alignment of nucleotide sequences guided by amino acid translations. Nucleic Acids Res.

[25] Wernersson R. (2003). RevTrans: multiple alignment of coding DNA from aligned amino acid sequences. Nucleic Acids Res.

[26] BLAST® Command Line Applications User Manual.

[27] Palatini U., Contreras C.A., Gasmi L., Bonizzoni M. (2022). Endogenous viral elements in mosquito genomes: current knowledge and outstanding questions. Curr Opin Insect Sci.

[28] Dezordi F.Z., Coutinho G.B., Dias Y.J.M., Wallau G.L. (2023). Ancient origin of Jingchuvirales derived glycoproteins integrated in arthropod genomes. Genet Mol Biol.

[29] Matthews B.J., Dudchenko O., Kingan S.B. (2018). Improved reference genome of aedes aegypti informs arbovirus vector control. Nature.

[30] Ter Horst A.M., Nigg J.C., Dekker F.M., Falk B.W., Pfeiffer J.K. (2019).

[31] Rombel I.T., Sykes K.F., Rayner S., Johnston S.A. (2002). ORF-FINDER: a vector for high-throughput gene identification. Gene.

[32] Katoh K., Rozewicki J., Yamada K.D. (2019). MAFFT online service: multiple sequence alignment, interactive sequence choice and visualization. Brief Bioinforma.

[33] Preston-Werner T. Semantic Versioning 2.0.0. Semantic Versioning. Accessed May 17, 2024. https://semver.org/.

[34] Loreto E.L.S., Melo E.S.D., Wallau G.L., Gomes T.M.F.F. (2023). The good, the bad and the ugly of transposable elements annotation tools. Genet Mol Biol.

