# Supplementary material for: EEfinder, a general purpose tool for identification of bacterial and viral endogenized elements in eukaryotic genomes
Source: Comput Struct Biotechnol J. 2024 Oct 18;23:3662–8. doi: 10.1016/j.csbj.2024.10.012 (PMC11532726; doi:10.1016/j.csbj.2024.10.012)
Supplement: Supplementary file 2 — Supplementary material [file mmc2.pdf]

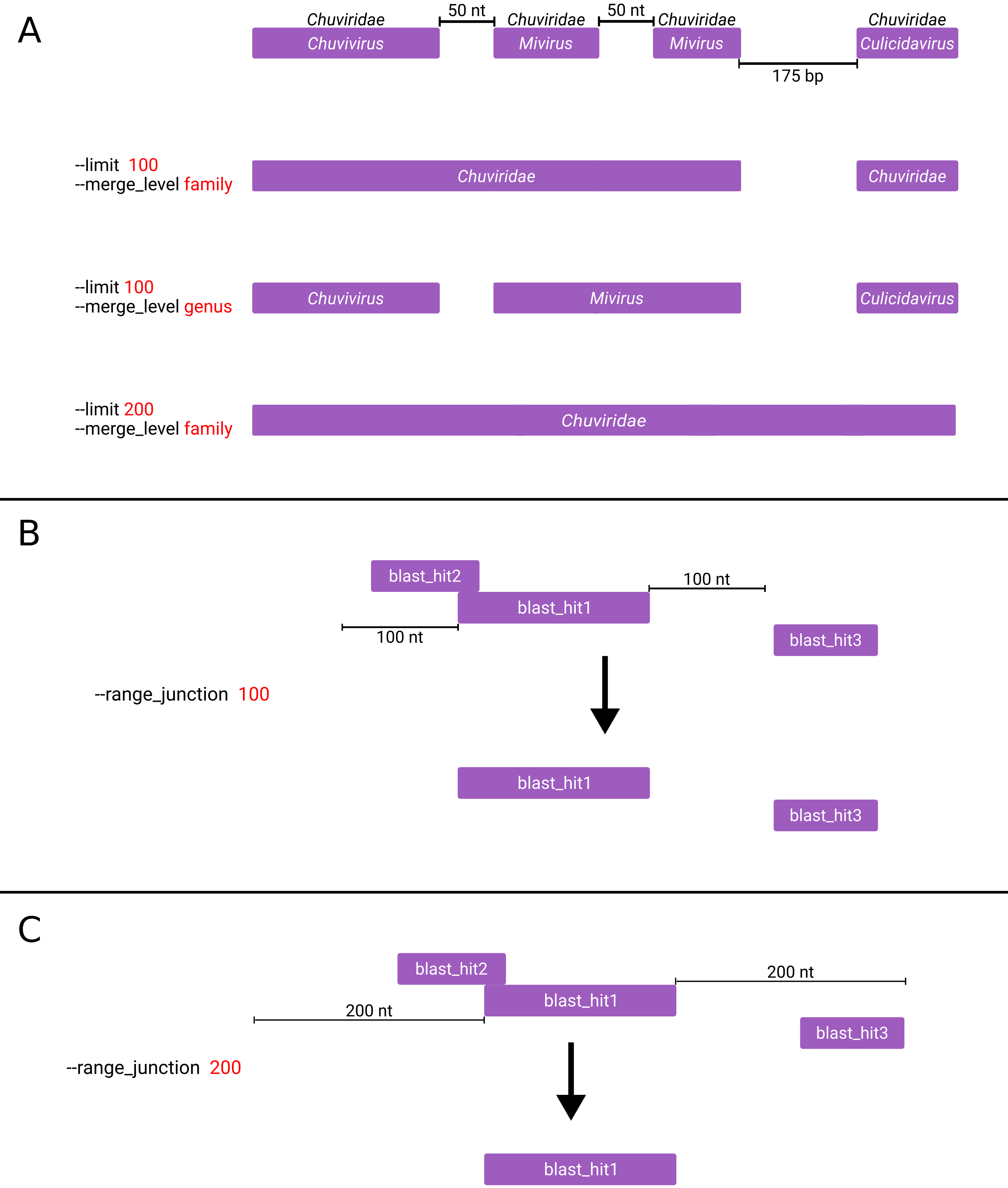

A. Demonstrates the execution of the ***--merge_level*** and ***--limit*** arguments with varying parameter values.
B. Illustrates the execution of ***--range_junction*** with a setting of 100 nt.
C. Illustrates the execution of ***--range_junction*** with a setting of 200 nt.
