# Supplementary material for: EEfinder, a general purpose tool for identification of bacterial and viral endogenized elements in eukaryotic genomes
Source: Comput Struct Biotechnol J. 2024 Oct 18;23:3662–8. doi: 10.1016/j.csbj.2024.10.012 (PMC11532726; doi:10.1016/j.csbj.2024.10.012)
Supplement: Supplementary file 3 — Supplementary material [file mmc3.pdf]

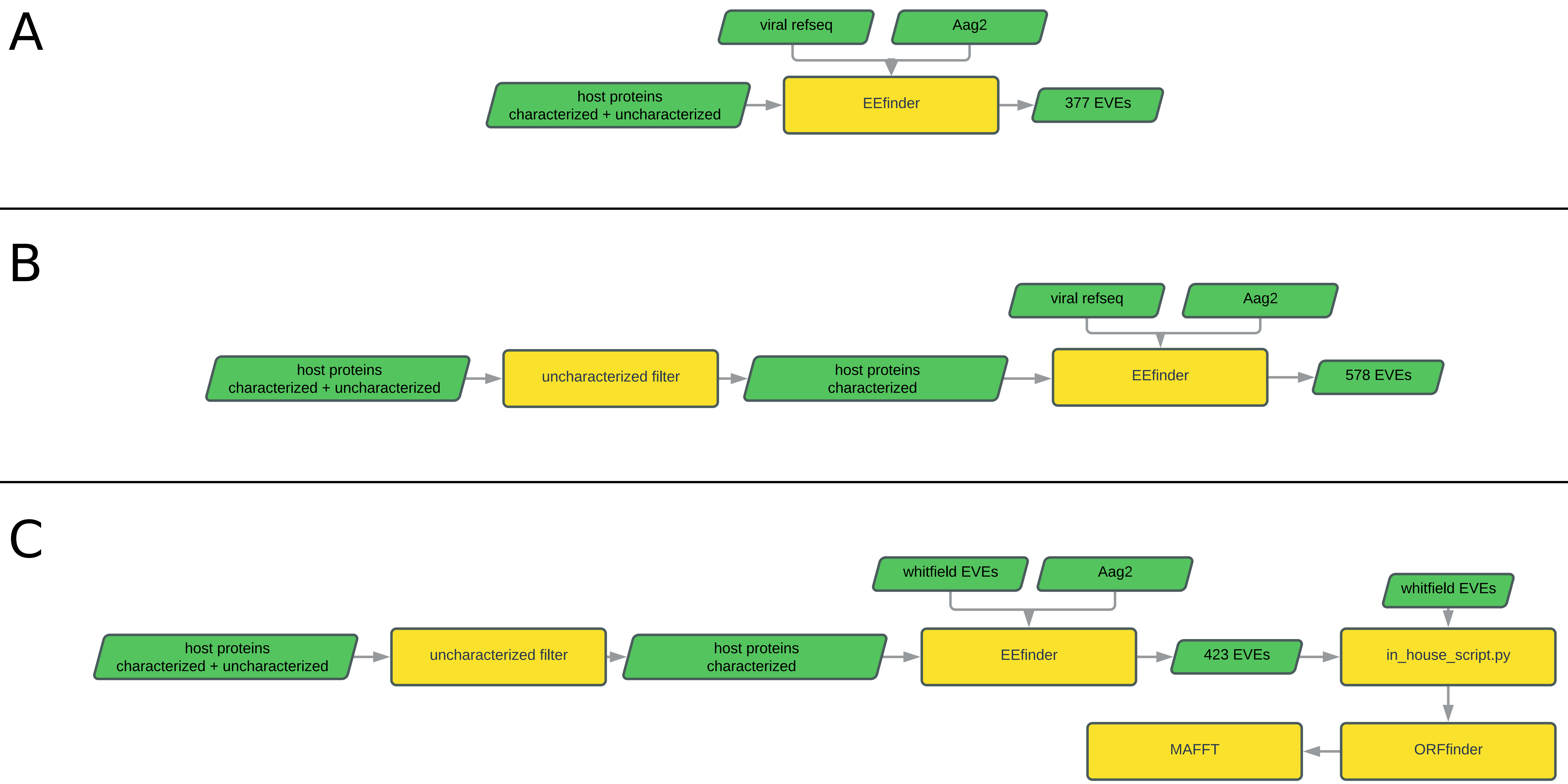

Workflow for endogenous viral elements validation section: **A.** EEfinder identified 377 EVEs using all Ae. aegypti proteins from NCBI RefSeq (updated September 8, 2022) as baits file, without any filtering. **B.** After filtering out "uncharacterized" and "hypothetical" proteins from the baits file, EEfinder identified 578 EVEs when analyzed against viral RefSeq proteins. **C.** Using the filtered baits file and Whitfield's EVEs as the viral reference, EEfinder detected 423 EVEs. These elements were then compared to Whitfield's EVEs using an in-house script, with ORFs extracted via ORFfinder and aligned using MAFFT.
