# Supplementary material for: EEfinder, a general purpose tool for identification of bacterial and viral endogenized elements in eukaryotic genomes
Source: Comput Struct Biotechnol J. 2024 Oct 18;23:3662–8. doi: 10.1016/j.csbj.2024.10.012 (PMC11532726; doi:10.1016/j.csbj.2024.10.012)
Supplement: Supplementary file 4 — Supplementary material [file mmc4.pdf]

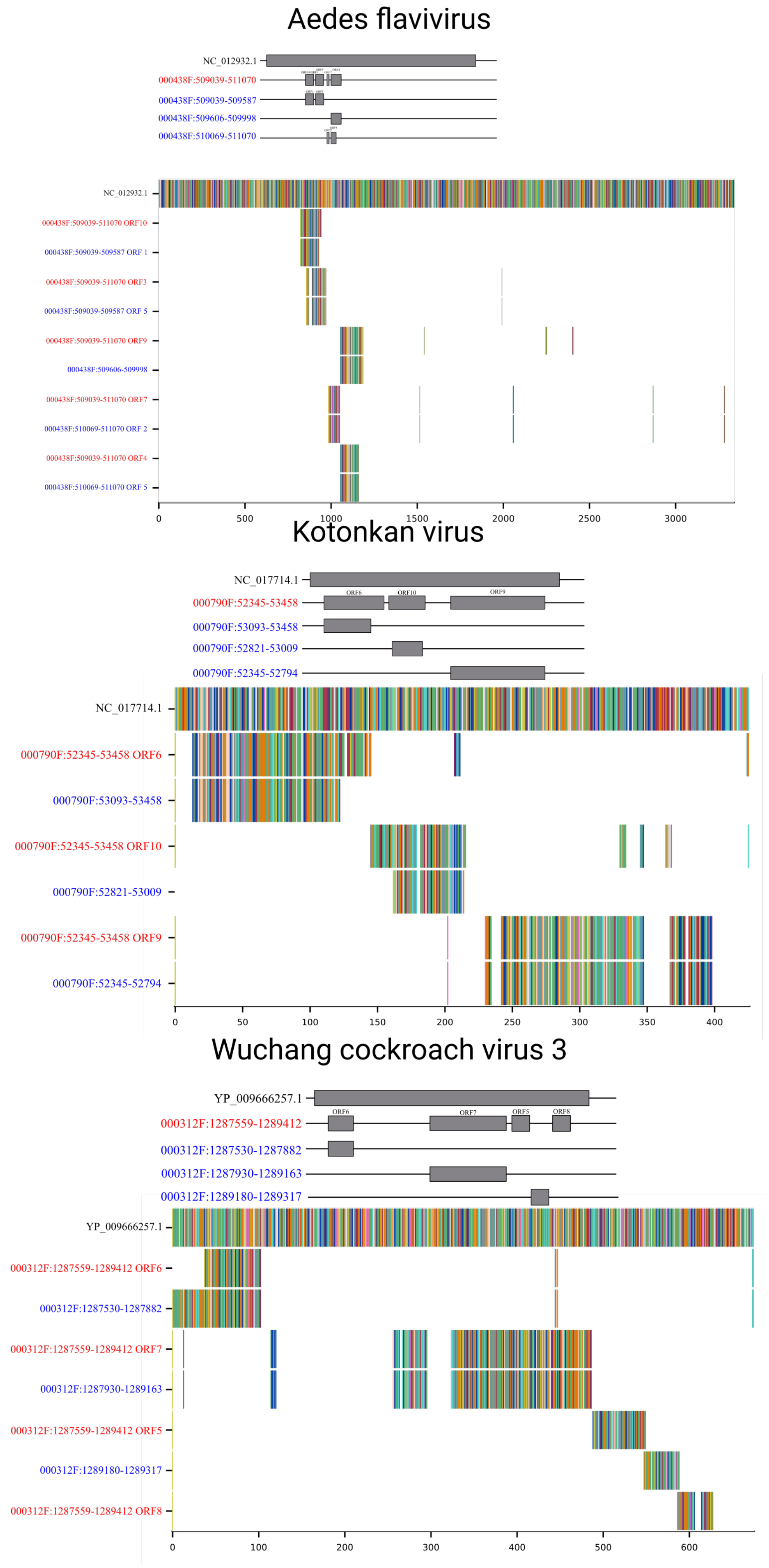

Multiple alignment showing the viral sequences corresponding to the endogenized region (in black font), the region identified by EEfinder (in red font), and the region described by Whitfield (in blue font).
