# Supplementary material for: EEfinder, a general purpose tool for identification of bacterial and viral endogenized elements in eukaryotic genomes
Source: Comput Struct Biotechnol J. 2024 Oct 18;23:3662–8. doi: 10.1016/j.csbj.2024.10.012 (PMC11532726; doi:10.1016/j.csbj.2024.10.012)
Supplement: Supplementary file 6 — Supplementary material [file mmc6.pdf]

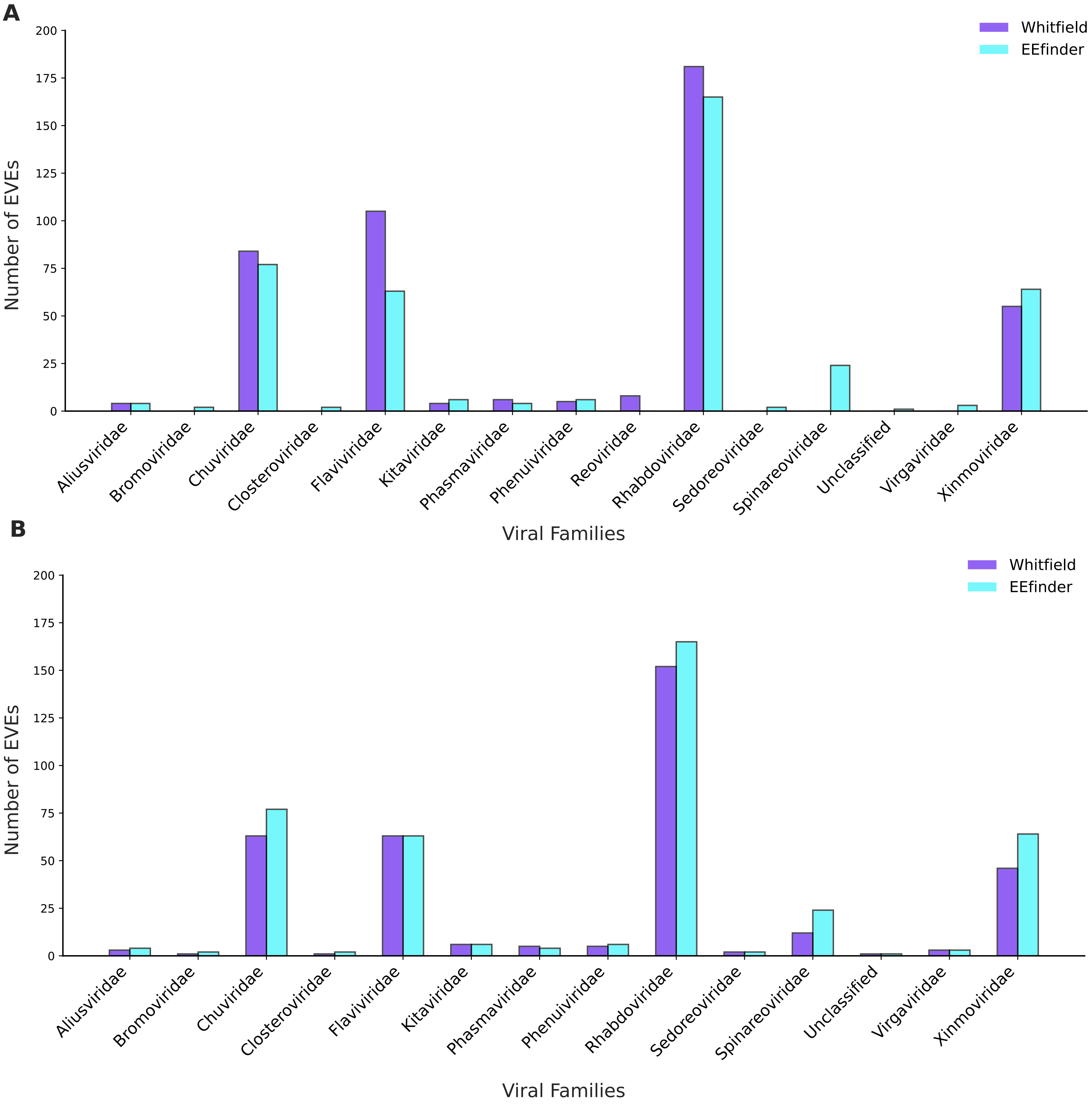

Barplots representing distribution of elements by viral families in each study.
A. Distribution of elements by family before the curation of degenerated regions.
B. Distribution of elements by family after the curation of degenerated regions.
