# Supplementary figures and images for: EEfinder, a general purpose tool for identification of bacterial and viral endogenized elements in eukaryotic genomes
Source: Comput Struct Biotechnol J. 2024 Oct 18;23:3662–8. doi: 10.1016/j.csbj.2024.10.012 (PMC11532726; doi:10.1016/j.csbj.2024.10.012)

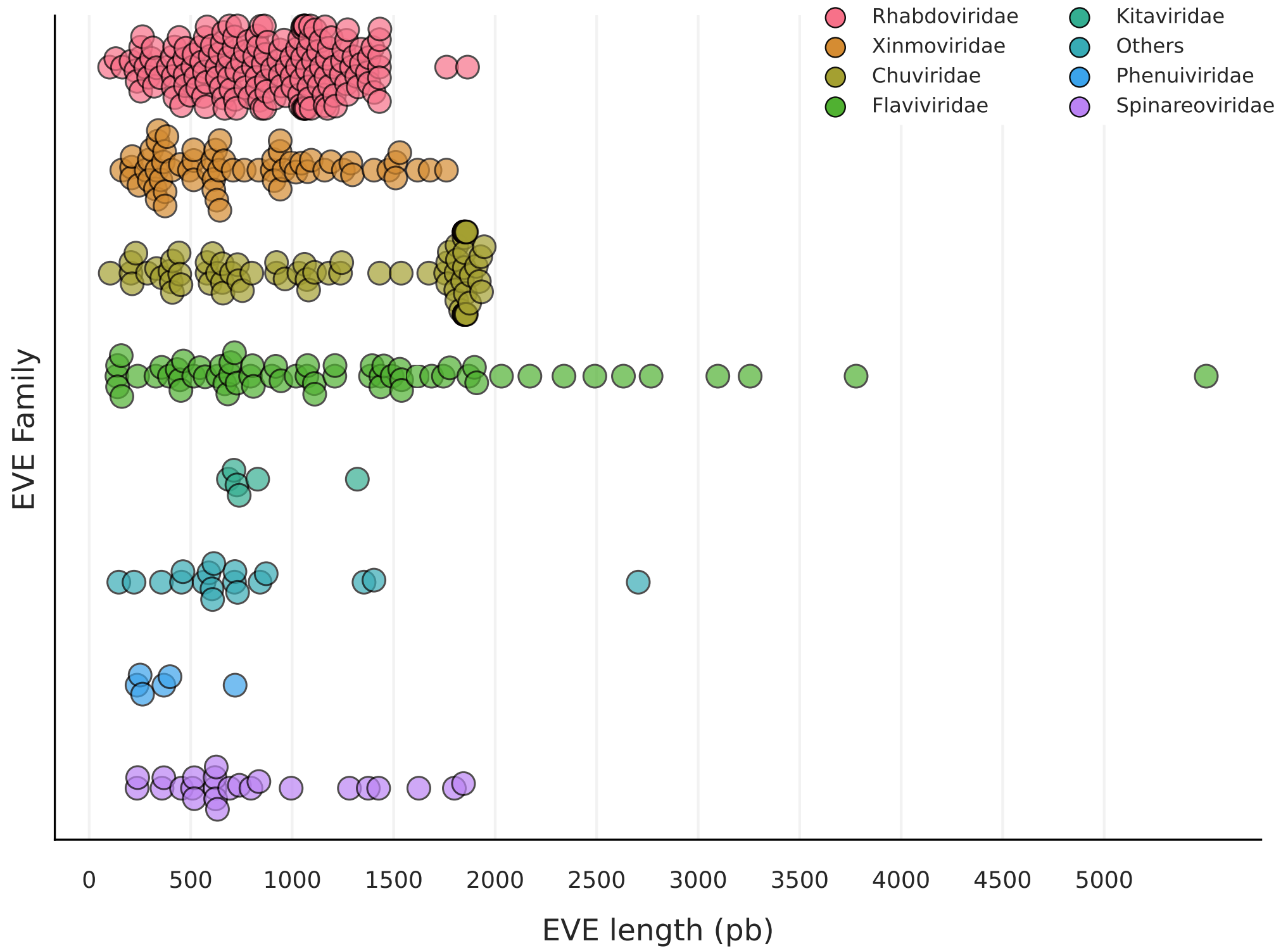

Beeswarm plot representing the distribution of EVEs by length and EVE family.

Supplement: Supplementary file 7 — Supplementary material [file mmc7.pdf]
