# Supplementary material for: EEfinder, a general purpose tool for identification of bacterial and viral endogenized elements in eukaryotic genomes
Source: Comput Struct Biotechnol J. 2024 Oct 18;23:3662–8. doi: 10.1016/j.csbj.2024.10.012 (PMC11532726; doi:10.1016/j.csbj.2024.10.012)
Supplement: Supplementary file 8 — Supplementary material [file mmc8.pdf]

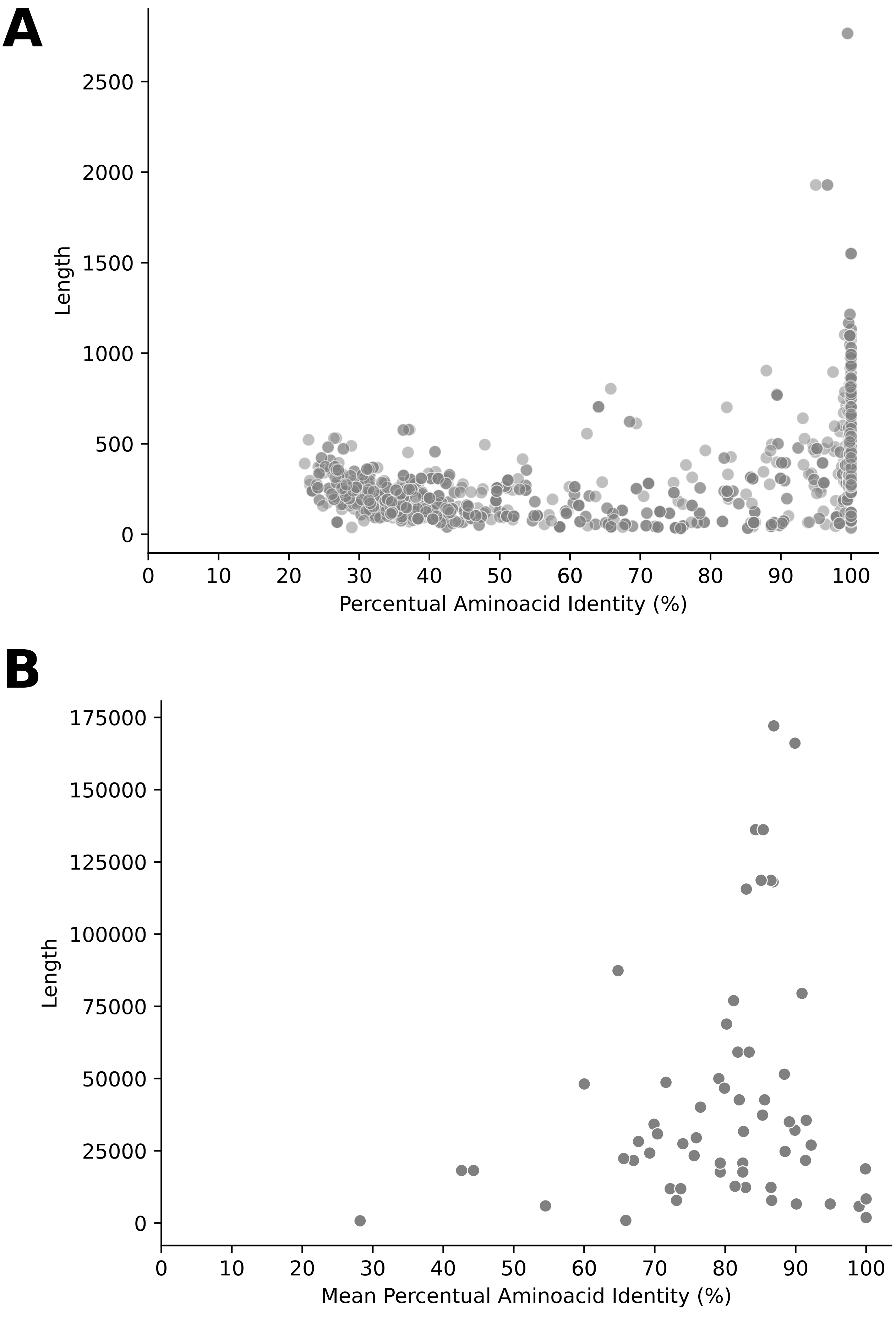

Comparison of percent amino acid identity and the length of wVulC endogenized regions found by EEfinder. **A.** Scatter plot of percent amino acid identity versus the length of endogenized regions before the EEfinder merge function. **B.** Scatter plot of mean percent amino acid identity versus the length of endogenized regions on scaffold 1 after the EEfinder merge function.
