# Supplementary material for: EEfinder, a general purpose tool for identification of bacterial and viral endogenized elements in eukaryotic genomes
Source: Comput Struct Biotechnol J. 2024 Oct 18;23:3662–8. doi: 10.1016/j.csbj.2024.10.012 (PMC11532726; doi:10.1016/j.csbj.2024.10.012)
Supplement: Supplementary file 9 — Supplementary material [file mmc9.pdf]

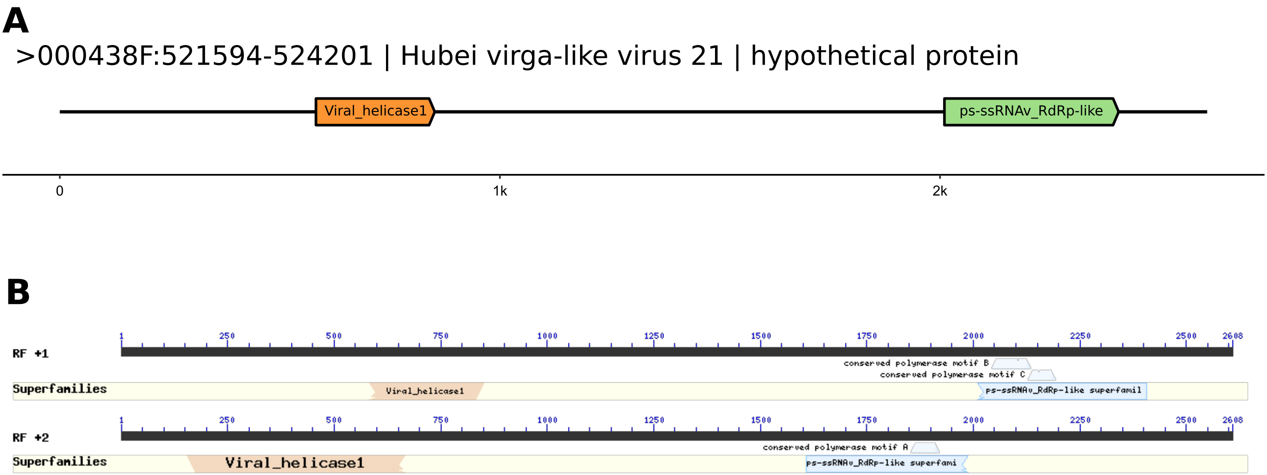

A. The EVE element classified as a hypothetical protein in NCBI RefSeq (YP_009337659.1), with a conserved helicase domain highlighted in orange and a conserved RNA-dependent RNA polymerase domain highlighted in green. The results were obtained using NCBI RefSeq viral proteins (updated on September 8, 2022) against the Aedes aegypti genome GCA_021653915 (Aag2). **B.** Conserved domain search results from NCBI CDD using the EVE element as the query.
